# Supplementary material for: Plasma Small Extracellular Vesicle Cathepsin D Dysregulation in GRN/C9orf72 and Sporadic Frontotemporal Lobar Degeneration
Source: Int J Mol Sci. 2022 Sep 14;23(18):10693. doi: 10.3390/ijms231810693 (PMC9504770; doi:10.3390/ijms231810693)
Supplement: Supplementary file 1 [file ijms-23-10693-s001.zip › ijms-1899683-supplementary.pdf]

Supplementary Material

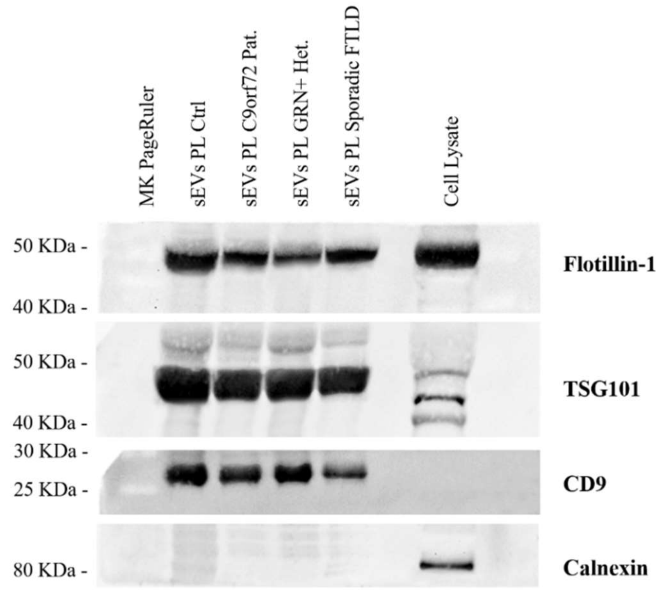

Figure S1. Plasma sEV characterization**.** Isolated plasma sEVs were CD9+ (a tetraspanin), TSG101+ and Flotillin-1+ (cytosolic proteins recovered in EVs) and Calnexin negative (endoplasmic reticulum residential protein, absent in EVs). Western Blot analysis on 4 representative samples (Ctrl, C9orf72 Pat., GRN+ Het., Sporadic FTLD) showed a decreasing trend in TSG101, Flotillin-1, and CD9 expression in sEVs from *C9orf72* pathological expansion carriers, *GRN* mutation carriers, and sporadic FTLD patients compared to controls.

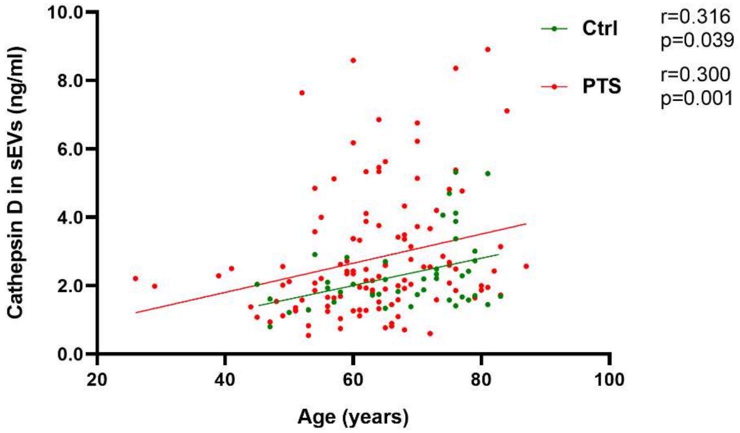

Figure S2. Correlation of total cathepsin D in sEVs and age. Correlation of total cathepsin D content in sEVs (ng/ml, not normalized values) with age in controls (Ctrl, in green) and patients (PTS, in red). Age was positively correlated with total cathepsin D in sEVs both in PTS (Spearman r=0.316, p=0.039) and in Ctrl (Spearman r=0.300, p=0.001). Spearman correlation r and p-values are shown in the panel.

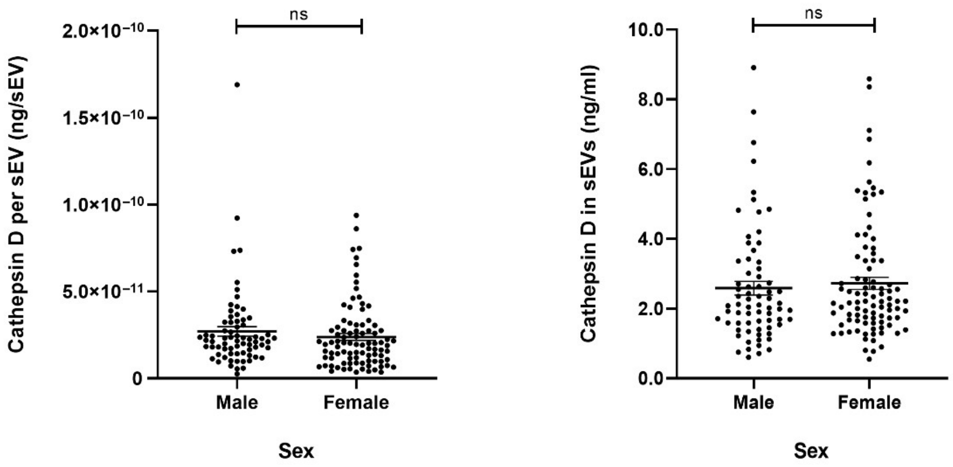

(a) (b)

Figure S3. No sex-related differences were observed in sEV cathepsin D levels. (**a**) Cathepsin D concentration per sEV (ng/sEV) and (**b**) total cathepsin D in sEVs (ng/ml) differences between male and females (p= 0.139 and p=0.470 respectively, Mann-Whitney test).

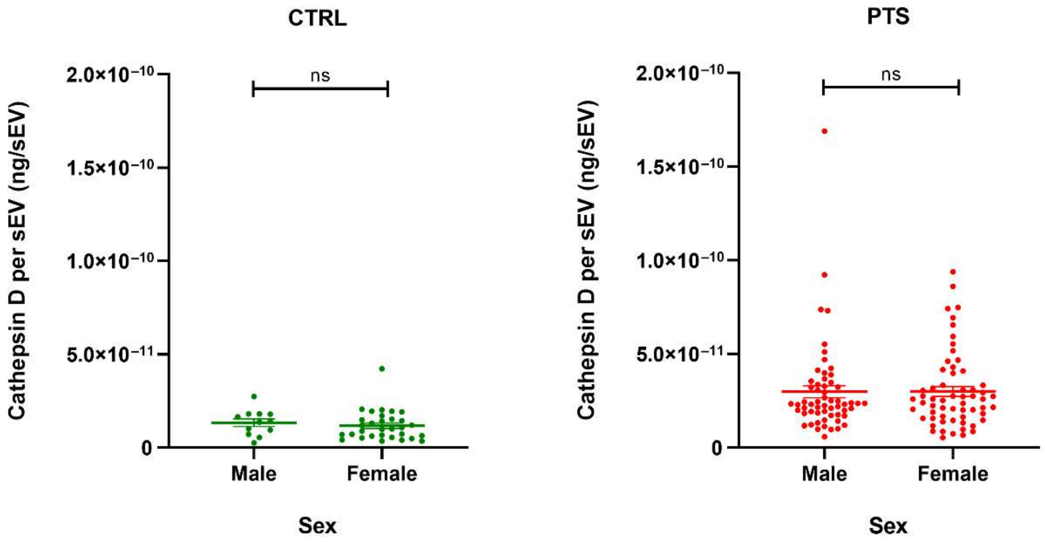

(a) (b)

Figure S4. No sex-related differences were observed in Cathepsin D concentration per sEV (ng/sEV) in (a) CTRL and (**b**) PTS between males and females (p= 0.543, Unpaired t-test and p=0.827, Mann-Whitney test, respectively).

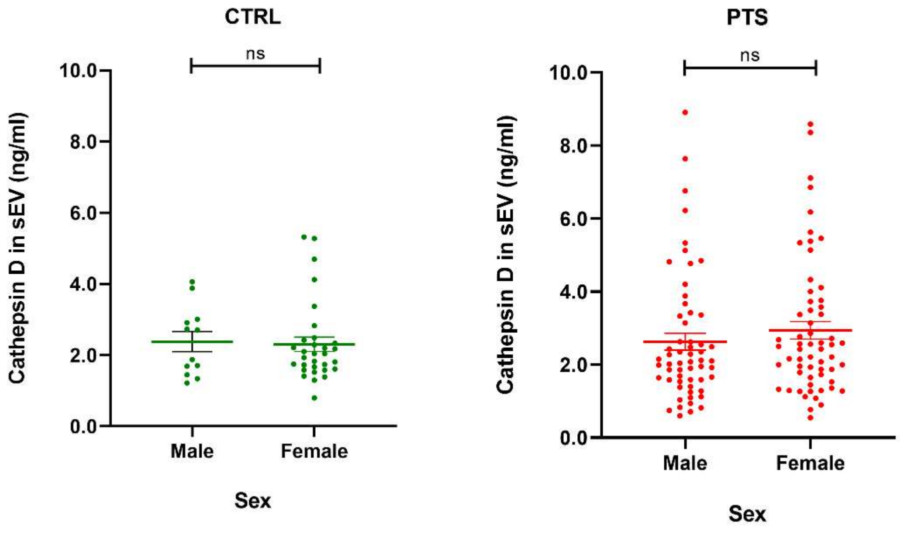

(a) (b)

Figure S5. No sex-related differences were observed in total cathepsin D in sEVs (ng/ml) in (a) CTRL and (b) PTS between males and females (p= 0.815 and p=0.208 respectively, Mann-Whitney test).

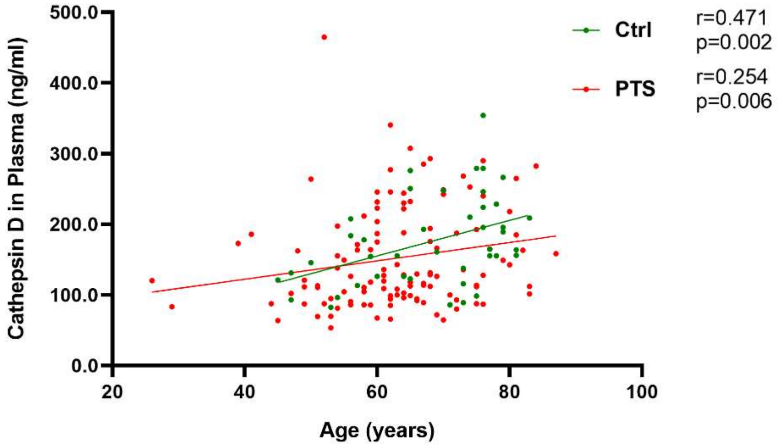

Figure S6. Correlation of cathepsin D concentration in plasma and age. Correlation of cathepsin D concentration in plasma (ng/ml) with age in controls (Ctrl, in green) and patients (PTS, in red). Age was positively correlated with plasma cathepsin D both in PTS (Spearman r=0.254, p=0.006) and in Ctrl (Spearman r=0.471, p=0.002). Spearman correlation r and p-values are shown in the panel.

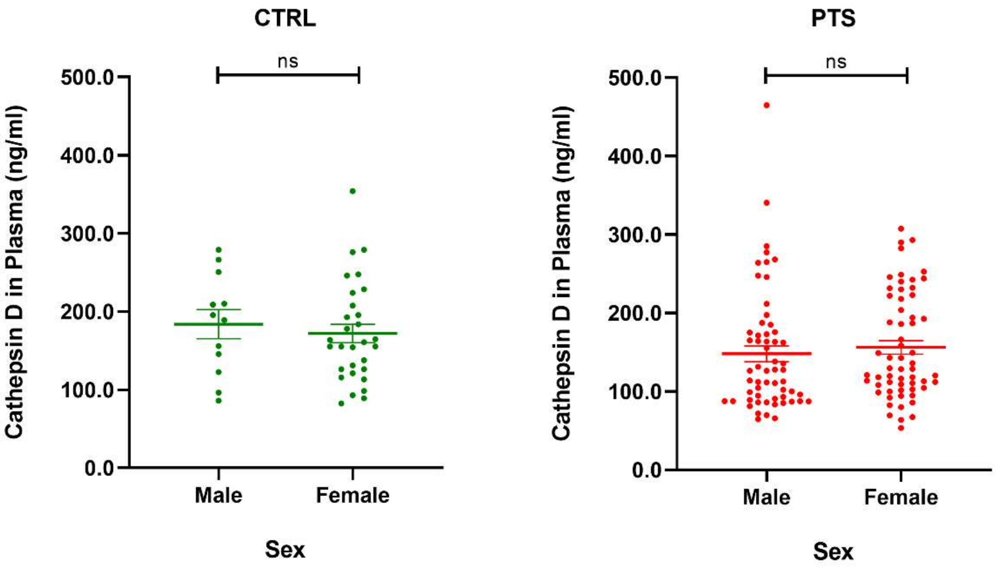

(a) (b)

Figure S7. No sex-related differences were observed in plasma cathepsin D (ng/ml) in (a) Ctrl and (b) PTS between males and females (p= 0.587 and p=0.238 respectively, Mann-Whitney test).

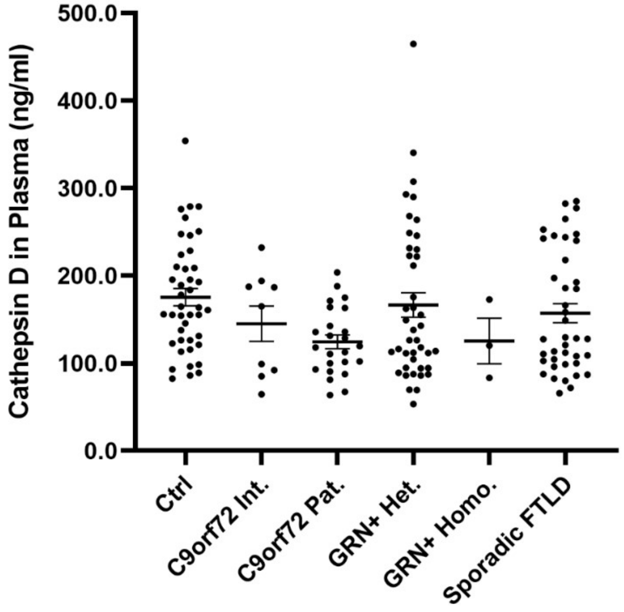

Figure S8. Cathepsin D concentration in plasma (ng/ml) in Ctrl, C9orf72 Int., C9orf72 Pat., GRN+ Het., GRN+ Homo., and Sporadic FTLD. Mean ± SEM. Dot plot represents raw data while the global p-value (p=0.055) was obtained by generalized linear model with inverse gaussian distribution adjusted for age and sex.

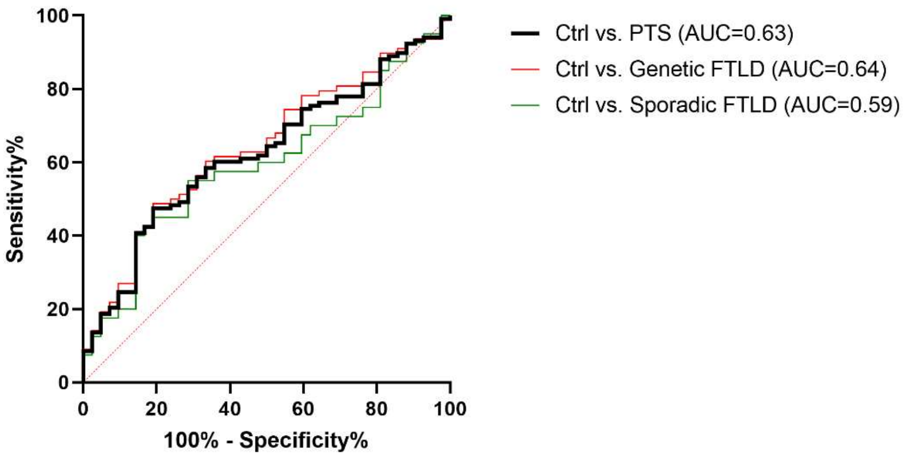

Figure S9. ROC curves for plasma cathepsin D. Plasma cathepsin D concentration was used to evaluate the discrimination of PTS from Ctrl. AUC Ctrl vs PTS 0.63 (black); AUC Ctrl vs Genetic FTLD 0.64 (red); AUC Ctrl vs Sporadic FTLD 0.59 (green). AUC comparison with DeLong test, p=0.565.
